# Happily Unhelpful: Infants’ Everyday Helping and its Connections to Early Prosocial Development

**DOI:** 10.3389/fpsyg.2018.01770

**Published:** 2018-09-21

**Authors:** Stuart I. Hammond, Celia A. Brownell

**Affiliations:** ^1^School of Psychology, University of Ottawa, Ottawa, ON, Canada; ^2^Department of Psychology, University of Pittsburgh, Pittsburgh, PA, United States

**Keywords:** prosocial behavior, infants, unhelpful helping, altruism, helping

## Abstract

Young children’s everyday helping in the home has received relatively little attention in research on prosocial behavior. Nevertheless, key features such as young children’s cheerful participation in chores around the home, including in ways that make accomplishing these chores more difficult for parents, can reveal important facets of early prosocial development. The present study reports the results of an Internet (MTurk) survey of over 500 families with children aged 1–4 years about their children’s prosocial tendencies, participation in nine common chores, whether children’s helping attempts were helpful or not, and attributions about children’s motives for helping. Consistent with much prior research, parents reported that children became more prosocial with age. The majority of parents reported children’s participation in everyday helping is at times unhelpful. Parents attributed children’s helping to a variety of motives and these too, changed with age. Fathers had somewhat different perceptions of children’s everyday helping than mothers. Results are discussed in terms of how understanding everyday helping can contribute to ongoing debates in the literature about the roots of prosocial behavior.

## Introduction

The fact that infants help others early in life, soon after the first birthday, if not earlier (e.g., Svetlova et al., 2010; Dahl, 2015; Hammond et al., 2017), may reveal something profound about human nature. Infants’ and toddlers’ efforts to help others, which exceed those of one our closest relatives, the chimpanzee, may suggest that humans have evolved a “hypercooperativeness” (Warneken and Tomasello, 2006, p. 1302; see also Vaish and Tomasello, 2014; Warneken, 2015). Many researchers in the field share Warneken and Tomasello’s (2006) view that humans are cooperative by nature. But important questions remain, such as whether prosociality is exclusively motivated by altruism (e.g., Hepach et al., 2012) or by other social motives (e.g., Carpendale et al., 2014; Pletti et al., 2017), and whether helping is unlearned, or if there is a role for socialization in prosocial development (e.g., Brownell and Early Social Development Research Lab, 2016; Warneken, 2016). Concerns about the role of evolution and development in children’s prosocial behavior also motivated earlier work in prosocial development (see, e.g., Bridgeman, 1983).

Seeking to elucidate these issues, in this paper we join a growing number of researchers in pointing out a mundane, but important, point: most human infants are not particularly good helpers. In experimental contexts, where infants are provided with opportunities to assist adults feigning distress, children often fail to help others (see Waugh and Brownell, 2017). In structured problem situations, infants often display wariness, turn to parents for security, or just continue to play. Infants’ inconsistent helping can also be seen in the descriptives of nearly any experimental study of helping. For example, Dunfield et al. (2011) study, no infants helped an experimenter who appeared to have hurt their knee, although some infants helped with other tasks. In Warneken and Tomasello’s (2006) study, in over half of the helping tasks fewer than half of the children helped. Despite the predominance of both helping and non-helping, when the time comes to draw conclusions from these studies, most often the overarching conclusion is simply that “toddlers help,” which they most certainly do – but why only sometimes? Looked at more closely, children’s actual behavior makes the claim that young children are reliably altruistic problematic when they often seem to care more about themselves than others; likewise, their behavior challenges claims that early helping is unlearned when this putative evolved mechanism seems to be built on an unsteady and unreliable foundation.

Moreover, even when they do try to help others, their behavior is often not helpful. In their daily lives, infants and toddlers engage in “everyday helping” as they participate in the life of the home, helping clean up toys, water the garden, and doing other tasks. But infants are not little angels, and parents do not look to raising them as a time of relative ease when they can relax while their young children help around the home and reduce their own workload. This fact was briefly raised in a seminal study by Rheingold (1982), who found that although toddlers can and do try to help, parents often “avoid what they viewed as interference … [by doing] chores while the children were taking their naps” (p. 122). Hammond (2014) labeled this phenomenon “unhelpful helping,” meaning that young children’s “helpful” participation makes the task more difficult for a parent rather than less. Although this construct has the potential to provide unique insights into the nature and motives for early-appearing prosocial behavior, no study, to date, has specifically examined “unhelpful helping:” how frequent is it, does it predominate earlier in the development of prosocial behavior, how does it relate to prosocial tendencies more generally?

More positively, Rheingold (1982) also noted that children take part in activities with others with good humor and sprightly energy. In this vein, Forman (2007) remarked that parents must sometimes manage toddlers who adamantly and enthusiastically want to participate in a given household task, whether the parent wants them to or not. As others have argued, toddlers have a strong motive to “belong” (Baumeister and Leary, 1995; Barragan and Dweck, 2014). Participating in parents’ activities fulfills that drive even without any helpful intentions. Ultimately, however, unhelpful toddlers become helpful, even caring, preschoolers. An important question is how children’s efforts and intentions to help change with age, from participating and playing to contributing and caring.

### Present Study

Although young children’s everyday helping in the home has received little attention in research on prosocial behavior, its features, such as the way children cheerfully participate in chores around the home, sometimes in ways that make these more difficult for their parents, may nevertheless reveal important characteristics about the structure of early prosocial development. In particular, systematically studying everyday helping in the early years can reveal how young children’s participation in such activities changes with age; to what extent their participation takes the form of interference or obstruction rather than helping; what motivates young children’s helping behavior in the home and how motives to help change with age. To examine these features of early helping we asked parents of 12 to 59 month old children to report their children’s current participation in everyday household chores, whether such participation was ever unhelpful, and what they believed motivated their children’s helpful and unhelpful participation.

## Materials and Methods

### Participants

Participants were recruited to and participated in the study through Amazon MTurk, and were compensated 50 cents for participation in what was an approximately 10-min anonymous survey. Participants needed to be living in the United States, 18 years of age and older, and the parent of a child between 1 and 4 years of age. If participants had two or more children of eligible age, they were asked to fill out the survey for the youngest eligible child.

Although MTurk is rarely used in developmental psychology research with young children, one of its advantages is that it draws a more diverse sample (see Buhrmeister et al., 2011). Indeed, unusually for a child development study, approximately half of the participants in the present study were fathers, the consequences of which we will discuss in more detail below.

#### Data Screening

Given that the data were collected nationally and anonymously, it was screened conservatively. Participants’ responses were eliminated if there were apparent mistakes in their data that might indicate inattention or falsification (e.g., inconsistencies in responses to the number of children living in the home, and the number of siblings the child has), and responses from multiple respondents were eliminated if coming from an identical IP address.

#### Final Sample

After screening, the final sample consisted of 528 participants (253 girls; 275 boys; 279 mothers; 249 fathers), with a mean age of *M*_age_ = 35.17 months (*SD*_age_ = 12.19 months), with children ranging from 12 to 59 months of age. Participants overwhelming identified their child as belonging to one ethnicity (86%), with approximately 14 percent reporting two or more ethnicities. Eighty-two percent of participants had some Caucasian ethnicity (68% Caucasian only), with approximately 12 percent of children being identified as primarily or some Hispanic or Latino (5% Latino Only), 11 percent as primarily or some African-American (7% Black only), 9 percent as primarily or some Asian (4% Asian only), and 2 percent as Native American or Pacific Islander (2% Native American or Pacific Islander only).

Approximately 10 percent of the children came from a household where the highest education attainment was a graduate degree, 38 percent of households held a bachelor degree, 14 percent of households held an associate degree, and 24 percent had some college, but no degree, 13 percent held a high school degree or equivalent, and about 1 percent had less than high school. Approximately 10 percent had incomes higher than 100,000 US dollars, 36 percent had a household income of between 50,000 and 99,999 US dollars, 40 percent of participants had a household income of between 25,000 and 49,999 US dollars, and approximately 14 percent had incomes below 25,000 US dollars.

### Procedures

Participants filled out an eligibility and consent form, then responded to a short set of questions on demographics, their child’s prosocial tendencies, and their child’s everyday helping in the home.

### Measures

#### Demographics

Participants were asked about their child’s gender and age; family income, education, and ethnicity as noted above; presence of siblings and pets in the home; and the child’s attendance at preschool or daycare.

#### Prosocial Tendencies

Participants filled out the prosocial subscale of the Goodman (1997) Strengths and Difficulties questionnaire, which comprised five questions about the child’s tendency to help and comfort others, on a three-point Likert scale ranging from Not True to Completely True. Responses were scored and summed to form a composite prosocial tendency score that ranged between 5 (Not True for all questions) to 15 (Completely True for all questions). The composite score had a Cronbach alpha of 0.76.

#### Everyday Helping

Participants were asked to fill-out a series of questions about their children’s help in the home.

#### Chores

Participants were asked about children’s participation in nine common chores in the home (laundry; vacuuming/sweeping; dishes; cooking/food preparation; groceries/shopping; gardening; putting away toys/cleaning up own room; throwing away trash). These common chores were derived from (unpublished) survey data collected with Hammond and Carpendale (2015). Responses were given a score of 1 if parents indicated that the chore was done Always/Almost Always or Sometimes, and 0 if done Rarely or otherwise, for a composite score that could range between 0 and 9. The composite score had a Cronbach alpha of 0.78. Parents could also fill-in an “Other” textbox to indicate other forms of helping.

#### Unhelpful Helping

Participants were asked to respond to the question “When your child gets involved in the above activities, is this ever unhelpful to you (e.g., they mix dirty laundry and clean laundry)? How do you respond in these sorts of situations?” Parents’ responses to these questions were scored with a 1 if they indicated the child was at times unhelpful (e.g., “Yes”; “I tell him what a good job he is doing, and when he’s not looking, I redo it”), and a 0 if they indicated the child was never unhelpful (e.g., “No”; “No, he is not unhelpful. He puts all of his toys away in his toy box, helps pick up the floor, and puts garbage in the trash can”).

#### Motives

Participants were asked “Why do you think your child wants to get involved in these sorts of activities?” and a series of six potential motives were listed: *being asked* (“Because I ask them to help”); *being rewarded* (“reward them when they help [e.g., sweets, allowance]”); *being praised* (“I praise them when they help”); *fun* (“They find these activities fun”); *social affiliation* (“They enjoy spending time with me”); and *care* (“They care about other people”). As with chores, these were drawn from a prior study (Hammond and Carpendale, 2015). Parents could check as many as applied, and responses were coded with a 1 if selected and a 0 if unselected. They were also afforded the option to fill out a text box with an “other” category for any other motives.

## Results

### Demographics in Relation to Prosocial Behavior

In preliminary analyses, age was related to several variables of interest, and subsequent analyses are broken down by children’s age in years. Parents’ gender was also related to several prosocial variables as noted and discussed further below. In contrast, children’s gender, and demographic variables such as household income and education, and reported ethnicity were unrelated to prosocial variables. For mean comparisons, non-parametric analyses (e.g., the Kruskal–Wallis test, an ANOVA analog) were used, as the number of participants by year of age was uneven.

### Prosocial Tendencies

The mean for parent-reported prosocial tendencies increased with children’s age, although the only significant difference was between Year 1, and all subsequent years (see **Table 1**; Kruskal–Wallis test, *p* < 0.001, with Dunn-Bonferroni *post hoc*, *p* < 0.001 for each *post hoc* comparison).

**Table 1:** Descriptives of prosocial tendencies, participation in chores, and unhelpful helping by age of child in years and gender of parents (with significant differences by parent gender noted).

	Prosocial tendencies		Chores		Unhelpful helping	
	Mothers	Fathers	Mothers	Fathers	Mothers	Fathers
All ages *N* = 528	11.93 (2.24)		4.08 (2.25)		0.76 (0.43)	
N_mother_ = 279	12.01	11.84	4.60^∗∗∗^	3.51	0.82^∗∗∗^	0.70
N_father_ = 249	(2.26)	(2.21)	(2.50)	(2.50)	(0.39)	(0.46)
Age 1 N_age1_ = 114	10.44 (2.34)		2.75 (2.33)		0.77 (0.42)	
N_mother1_ = 65	10.35	10.55	2.86	2.60	0.83	0.69
N_father1_ = 49	(2.58)	(2.11)	(2.17)	(2.53)	(0.38)	(0.47)
Age 2 N_age2_ = 126	12.20 (2.24)		3.98 (2.68)		0.76 (0.43)	
N_mother2_ = 65	12.35	12.05	4.51^∗^	3.42	0.82	0.71
N_father2_ = 61	(2.23)	(2.25)	(2.61)	(2.67)	(0.39)	(0.46)
Age 3 N_age3_ = 201	12.25 (1.90)		4.33 (2.44)		0.76 (0.43)	
N_mother3_ = 93	12.39	12.13	5.10^∗∗∗^	3.67	0.82^∗^	0.70
N_father3_ = 108	(1.76)	(2.01)	(2.31)	(2.35)	(0.38)	(0.46)
Age 4 N_age4_ = 87	12.77 (1.90)		5.40 (2.06)		0.75 (0.44)	
N_mother4_ = 56	12.93	12.48	5.88^∗∗^	4.55	0.79	0.68
N_father4_ = 31	(1.63)	(2.32)	(1.85)	(2.17)	(0.41)	(0.48)

^∗^*p < 0.05, ^∗∗^*p* < 0.01, ^∗∗∗^*p* < 0.001*.

### Everyday Helping

#### Chores

**FIGURE 1:**
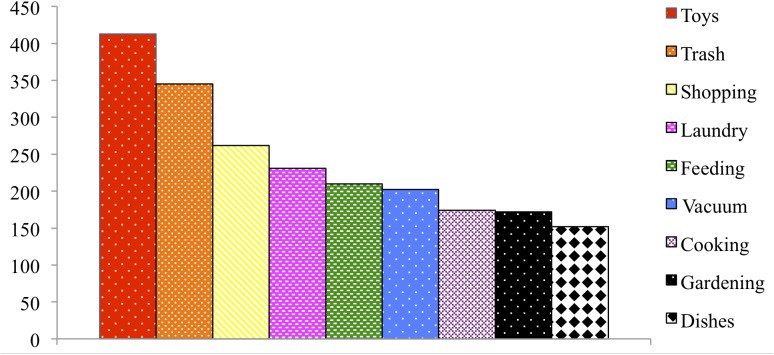
Number of children participating in chores at least sometimes across ages 1 through 4.

**Figure 1** depicts the most commonly reported chores across ages. The mean number of chores that children participate in increased with children’s age (see **Table 1**; Kruskal–Wallis test, *p* < 0.001). Participation in chores at Year 1 differed from all subsequent years (Dunn-Bonferroni *post hoc*, *p* < 0.001 for each *post hoc* comparison), participation at Year 2 and Year 3 did not differ from each other (Dunn-Bonferroni *post hoc* comparison, n.s.), and participation at Year 2 and Year 3 both differed from Year 4 (Dunn-Bonferroni *post hoc* comparison, *p* < 0.001). **Table 2** displays some examples of other types of helping that parents provided in the fill-in section for “other helping.”

**Table 2:** Individual examples of helping and parental views on unhelpful helping.

	Example
Other forms of helping	“She loves to wipe the refrigerator clean.” – Mother of 21 month-old girl
	“She tries to help me apply makeup. If I need something from another room, she’ll want to get it. She likes helping washing cars. She helps me turn on the Apple TV.” – Mother of 44 month-old girl
	“Telling me when something is wrong, closing doors for me, opening doors for me, helping me carry things.” – Mother of 50-month-old boy
	“Helps to find lost objects, like TV remote, car keys, shoes, etc.” – Father of 26 month-old girl
	“Answer phones. Taking care of someone who is sick. (get blanket, crackers, water)” – Father of 39 month-old girl
	“Getting diapers for his little sister, and choosing clothes for the day.” – Father of 49-month-old boy
Responses to unhelpful helping	“… Throwing away trash, she doesn’t always put the things she should in the trash can or she will put them in and take them back out again. We just have to make sure the trash goes in and stays there. Also, we have to check several times a day to make sure that she hasn’t put things in the trash can that don’t go in the trash. We believe she lost one of her favorite shoes that way.” – Mother of 14 month-old girl
	“Cooking is always an adventure. Recipes might not turn out to be 100% accurate but she’s learning so I don’t care too much about it. Cooking makes her happy so it makes me happy as well.” – Mother of 21 month-old girl
	“Yes it is usually unhelpful and makes everything take longer, but she loves learning new things and it is my job as mommy to teach her all of these things.” – Mother of 32 month-old girl
	“He sometimes sprays too much cleanser while dusting, I have to remind him it only needs a spray or two and he’ll stop (but forget the next time).” – Mother of 51-month-old boy
	“Sometimes she gets in the way of gardening or steps on plants. I just explain to her that she is hurting the plant and she normally stops.” – Father of 38-month-old girl
	“My son throws away his own trash when I ask him. Which is helpful. He likes to help me in the garden, which is a mixed bag. He always goes to the grocery store with me. Which is neither helpful nor unhelpful. He feeds our cat which is helpful every morning before daycare. He loves to help me vacuum which he honestly just sort of gets in the way but I think its good that he is attempting to help.” – Father of 48-month-old boy

Children’s reported participation in chores also varied by parents’ gender (see **Table 1**). Mothers reported greater participation in chores across ages than fathers (Mann–Whitney, *p* < 0.001), and, analyzed by year, this difference was significant at Year 2 (Mann–Whitney, *p* < 0.05), Year 3 (Mann–Whitney, *p* < 0.001), and Year 4 (Mann–Whitney, *p* < 0.01).

#### Unhelpful Helping

Across ages, the majority of parents reported their children engaged in unhelpful helping. See **Table 2** for examples of unhelpful helping. Parent-reported unhelpful helping did not increase by age (see **Table 1**). Across all ages, mothers reported more unhelpful helping than fathers (Mann–Whitney, *p* < 0.001); broken down by age, this parental gender difference was significant only at Age 3 (Mann–Whitney, *p* < 0.05).

#### Motives

**FIGURE 2:**
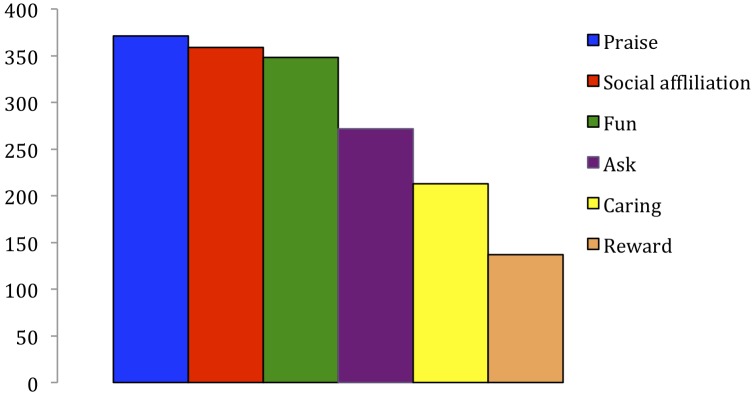
Frequency of reported motivations for everyday helping across ages 1 through 4.

Across ages, parents endorsed praise as the most likely motive for children’s helping, followed by fun, social affiliation, being asked, caring for others, and being rewarded (see **Figure 2**). Overall, mothers were more likely to endorse praise (Mann–Whitney, *p* < 0.01) and fun (Mann–Whitney, *p* < 0.01) as motives for children’s participation than fathers. Although most parents left the other text box for motives empty, a notable minority endorsed imitation, mimicry, and copying parents as a motive for helping (e.g., “He wants to be just like mommy”; “I really think it is because they like to be like us as much as possible”).

##### Motives by year

**FIGURE 3:**
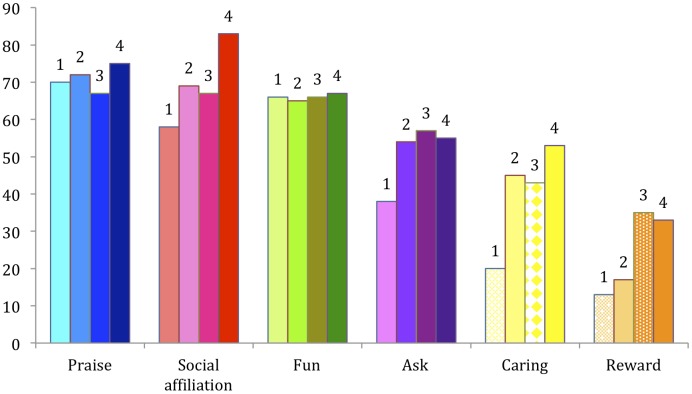
Percentage of parents attributing being asked, being rewarded, being praised, fun, social affiliation, or caring as motivations for children’s help by age in years.

Broken down by age, the most frequently endorsed motivation at Year 1 and Year 2 was praise, with a tie between being praised and social affiliation at Year 3. Social affiliation was the most frequently endorsed motive at Year 4 (see **Figure 3**).

##### Motives by type by year

Parents tended to endorse more motives as children grow older (Kruskal–Wallis test, *p* < 0.001; also, see **Figure 3**), though *post hoc* tests suggest that this difference lies in the difference between children at Year 1 versus all other years (Dunn-Bonferroni *post hoc*, *p* < 0.05 for Year 1 to Year 2, *p* < 0.01 for Year 1 to Year 3, and *p* < 0.001 for Year 1 to Year 4), though there were no differences between Year 2, 3, or 4. In Year 1, over half of parents endorsed three motives for children’s helping (praise; fun; social affiliation). By Year 4, over half of parents endorsed five motives for children’s helping (social affiliation; praise; fun; being asked; and caring for others).

Rates of parental endorsement of fun and praise as motives for helping did not differ by year. In terms of values that did differ significantly, parents were more likely to endorse being asked as a motive for children’s helping at Year 3 than Year 1 (Kruskal–Wallis test, *p* < 0.05, with Dunn-Bonferroni *post hoc*, *p* < 0.01). Parents were more likely to endorse being rewarded at Years 3 and 4 than Year 1 (Kruskal–Wallis test, *p* < 0.001, with Dunn-Bonferroni *post hoc*, *p* < 0.001 for Year 1 and Year 3, and *p* < 0.01 for Year 1 and Year 4), and at Year 3 than Year 2 (Dunn-Bonferroni *post hoc*, *p* < 0.01). Parents were more likely to endorse social affiliation at Year 4 than Years 1 or 3 (Kruskal–Wallis test, *p* < 0.01, with Dunn-Bonferroni *post hoc*, *p* < 0.01 for Year 1 and Year 4, and *p* < 0.05 for Year 3 and Year 4). Parents were more likely to endorse caring at Years 2 through 4 than at Year 1 (Kruskal–Wallis test, *p* < 0.001, with Dunn-Bonferroni *post hoc*, *p* < 0.001 for each *post hoc* comparison).

### Relations Between Prosocial Tendencies, Everyday Helping, and Motives for Helping

**Table 3** depicts partial correlations (Spearman’s rho) between prosocial tendencies, participation in chores, unhelpful helping, and parent-reported motives for helping, controlling for age in months. Parents’ gender was not controlled in this analysis, as for all means, mothers had a consistently higher value than fathers, therefore correlations were relatively unchanged, and due to concerns with including a dichotomous variable as a control variable (though see Bay and Hakstian, 1972). Children’s participation in chores was correlated with both their prosocial tendencies (0.30, *p* < 0.001) and unhelpful helping (0.22, *p* < 0.001), but prosocial tendencies and unhelpful helping were unrelated (n.s.).

**Table 3:** Partial Spearman’s rho correlations, controlling for age in months, between children’s prosocial tendencies, participation in chores, unhelpful helping, and attributed motivations for helping.

	Prosocial tendencies	Chores	Unhelpful helping
Chores	0.30^∗∗∗^	–	–
Unhelpful helping	-0.04	0.22^∗∗∗^	–
Ask	0.02	0.14^∗∗^	0.01
Reward	0.08	0.07	-0.01
Praise	0.12^∗∗^	0.21^∗∗∗^	0.14^∗∗^
Fun	0.20^∗∗∗^	0.32^∗∗∗^	0.14^∗∗^
Social affiliation	0.15^∗∗^	0.27^∗∗∗^	0.12^∗∗^
Caring	0.36^∗∗∗^	0.26^∗∗∗^	0.08

^∗∗^*p < 0.01; ^∗∗∗^*p* < 0.001*.

Praise, fun, social affiliation, and caring were all related to children’s prosocial tendencies, with helping motivated by caring being the best predictor of prosocial tendencies (0.36, *p* < 0.001). All motives were related to participation in chores, except for reward, with everyday helping motivated by fun being the best predictor of participation in chores (.32, *p* < 0.001). Praise, fun, and social affiliation were related to unhelpful helping, with helping motived by praise and fun being the best predictors of unhelpful helping (both at 0.14, *p* < 0.01).

## Discussion

The present study reported on the findings of a survey of over 500 participants, examining parental reports of prosocial tendencies and everyday helping among children aged 1–4 years (12–59 months), including participation in common chores, tendency to be unhelpful during chores, and motives for everyday helping.

### Developmental Trends in Prosocial Behavior

As with prior studies, children generally become more helpful with age (e.g., Svetlova et al., 2010), with greater prosocial tendencies, and greater proclivity toward everyday helping. Also supporting prior studies, children participate in some forms of help quite early in life, even by about 12 months of age (e.g., Dahl, 2015; Hammond et al., 2017).

Controlling for age, children with higher prosocial tendencies had a higher tendency to participate in chores. However, whereas caring as a motive for everyday helping best predicted children’s prosocial tendencies, fun as a motive best predicted participation in chores. This offers partial support to Dunfield’s (2014) proposition that different domains of prosocial behavior have unique developmental pathways. More importantly, it shows that motives for everyday helping become more complex, as well as more numerous, with development, with praise and fun as the foundation of early helping, and social affiliation and caring emerging to greater prominence later.

### Everyday Helping and Unhelpful Helping

Supporting Rheingold’s (1982) finding that children’s assistance can be a nuisance for parents, the majority of parents in the present study reported that their children’s help in the home is at times unhelpful. This finding is somewhat different than that of Waugh and Brownell (2017), who studied the frequent non-helping behaviors that emerged in toddlers’ interactions with unfamiliar adults experiencing difficulties in laboratory tasks. In the home, children are often explicitly attempting to participate (i.e., they are not warily backing away from their parents), even when the parent is not experiencing difficulty in completing the task (and if they are, it is often the child’s participation that is making it more difficult). That is, “helping” in the home is often not about helping someone else with a difficulty, but about participating or collaborating together in the same activity.

Although unhelpful helping seems to be unrelated to children’s general prosocial tendencies, it is related to participation in chores in the home. Parents who were more likely to report their children’s motives for helping as fun, social affiliation, and praise were also more likely to report unhelpful helping. Parents in the present study noted diverse ways of managing unhelpful helping. One notable approach was to view the unhelpful behavior as part of positive development that would one day lead to genuinely helpful behavior, or to appreciate the behavior as pleasurable, enjoyable, or amusing. This finding supports Brownell et al. (2002) and Brownell and Early Social Development Research Lab (2016) contention that positive social interactions form an important context for prosocial development. These strategies may also support Rogoff and colleagues’ (e.g., Rogoff, 2014) findings that European Americans treat their infants’ efforts to help others in “mock” ways, in the sense that the efforts are seen as cute and pleasant, but not necessarily important contributions to the life of the family or community. In contrast, in many indigenous communities, children come to play serious and important roles in supporting community living.

### Motives for Helping

The present study found that parents endorsed both internal (care; fun; social affiliation) and external (being asked; being praised; being rewarded) motivators for children’s help. Supporting the general view in the literature that internal motives are of greater interest and importance (e.g., Rheingold, 1982; Warneken and Tomasello, 2006; Hepach et al., 2012), being asked and particularly being rewarded were less predictive of prosocial tendencies and helping. Further supporting Rheingold (1982) and others (e.g., Carpendale et al., 2014), parents attribute multiple motives for their children’s helping, predominantly praise, social affiliation, and fun. Fun and social affiliation seemed to be important motivators for everyday helping across ages, supporting Rheingold’s (1982) view that children’s help is often characterized by cheerful energy, or “alacrity.”

However, contrary to a wholly internally motivated view of helping, parents tended to endorse praise as a motivator for helping (see also Dahl, 2015). Parents’ ranking of praise as a primary motivator for helping, and particularly its presence at younger ages, is important to the extent that this would suggest that parents often use praise (and indeed, this emerges in parents’ characterization of managing even unhelpful helping, e.g., “Yes it is usually unhelpful, but I praise him for helping anyway while correcting” – Mother of 23-month-old boy). The widespread use of praise even with the youngest children weakens support for at least one argument for early helping being unlearned, namely that “[i]nfants 18 months of age are too young to have received much verbal encouragement for helping from parents” (Warneken and Tomasello, 2006, p. 1302).

The findings reported here present somewhat mixed support for the view that caring, often seen as a form of altruism, is foundational to the early emergence of prosocial behavior. Overall, parents endorsed caring as a motivator for everyday helping less frequently than many other motives. However, care seems to emerge as a more important motivator of helping in older children, suggesting that this feature of prosocial behavior is later developing, building on earlier parental praise and having fun as motives. With age controlled, care does seem to predict children’s prosocial tendencies and their helping in the home, although not unhelpful helping, showing that altruistic motives are important to prosocial behavior, consistent with the assertions of many theories.

### Fathers’ Perceptions of Children’s Prosocial Behavior

The present study had an unusually high number of fathers, approximately half the sample, likely because it was administered via MTurk. As participants in most studies of prosocial behavior are mothers, we had few expectations of how fathers’ responses might differ from those of mothers. Fathers appear to view their children’s everyday helping and unhelpful helping as occurring at lower rates than do mothers, although both mothers and fathers seem to view children’s prosocial tendencies in about the same way. Fathers are also less likely to see fun and being praised as motives for everyday helping. Although the issue is impossible to resolve from the present data, one possibility is that mothers are spending more time with their young children, or do more chores in the home to begin with, and are more likely to notice children’s helping, and unhelpful helping.

### Limitations

This is the first study of early helping to use MTurk to obtain information from a wide swath of parents about their young children’s behavior. However, survey data have many well-known weaknesses, relying heavily on parents’ perceptions of their children’s helping, rather than direct observation. However, we hope that the present study will encourage researchers to explore everyday and unhelpful helping in future observational and experimental studies.

Although the participants in the present study are more diverse than a typical developmental study, particularly in terms of parental gender, the participants are nevertheless largely from Caucasian majority ethnic backgrounds, and may respond differently to children’s help than other parents (e.g., Rogoff, 2014). Given the unexpected findings about fathers’ perceptions of prosocial behavior, more details about primary parenting, time spent with children, and the parents’ involvement in the life of the home would be advisable in future research studies.

### Social Ecology and Future Directions

This study began by noting a consensus in the research literature on prosocial development, namely that human infants are hypercooperative compared to other species. The findings presented here add to recent research that explores what Dahl (2017) calls the ecology of development, and presents evidence that challenges some evolutionary speculations in the field, such as to natural altruism and unlearned helping. Children may begin helping with motives other than altruism, such as fun and social affiliation (see Rheingold, 1982; Pletti et al., 2017), and seem to assist others in ways that are not always very helpful (see also Waugh and Brownell, 2017). In noting these disruptive developmental realities, we wish to close by offering a further, if speculative, evolutionary hypothesis for the field to explore.

The present study found evidence, also suggested by other researchers (e.g., Rheingold, 1982; Carpendale et al., 2014; Brownell and Early Social Development Research Lab, 2016), that positive contexts are important to prosocial development. Children may be motivated to engage in everyday helping by a desire for fun, and their efforts are often unhelpful. Yet at an evolutionary level of analysis, *play* is an important evolved feature of mammalian behavior, and human infants seem to be the most playful of all (see Bruner, 1972; Schank et al., 2015). Play helps animals learn about and explore their environment and social others in ways that are, to the outside observer, often quite silly to behold. As we consider the role of positive, and even fun, contexts and the role of unhelpful helping in early prosocial development, perhaps we should also begin to conceptualize humans’ evolved nature as “hyperplayful” as well as hypercooperative.

## Ethics Statement

This study was carried out in accordance with the recommendations of the Institutional Review Board, at the Research Conduct and Compliance Office, University of Pittsburgh. The protocol was approved by the University of Pittsburgh Institutional Review Board. All subjects gave written informed consent in accordance with the Declaration of Helsinki.

## Author Contributions

SH was the primary author. CB contributed to research, conceptualization, editing, and analysis.

## Conflict of Interest Statement

The authors declare that the research was conducted in the absence of any commercial or financial relationships that could be construed as a potential conflict of interest. The reviewer RB and handling Editor declared their shared affiliation at the time of review.
